# Background factors associated with academic motivation for attending medical school immediately after admission in Japan: A single‐center study

**DOI:** 10.1002/jgf2.528

**Published:** 2022-02-16

**Authors:** Takashi Watari, Nobuhiro Nagai, Kaori Kono, Kazumichi Onigata

**Affiliations:** ^1^ General Medicine Center Shimane University Hospital Shimane Japan; ^2^ Shimane University Faculty of Medicine Shimane Japan; ^3^ Postgraduate Clinical Training Center Shimane University Hospital Shimane Japan

**Keywords:** academic motivation, amotivation, medical school, professionalism, self‐determinant motivation

## Abstract

**Background:**

To become a doctor with a high level of professionalism and ethical standards, it is important to have and maintain a high level of motivation right from medical school. However, studies in Japan have not quantitatively investigated the factors related to motivation immediately after enrollment. This study aimed to identify the demographic factors that influence the motivation of medical students immediately after admission.

**Methods:**

A cross‐sectional single‐center study was conducted. First‐year medical students answered our questionnaire three weeks after the admission. The questionnaire comprised 16 demographic items and the 28‐item Academic Motivation Scale, which was used to quantify motivation.

**Results:**

Our analysis showed that amotivation, representing low levels of self‐determinant motivation, was significantly higher in students whose parents were medical professionals and in students who did not talk about their problems than in those whose parents were not medical professionals and those who did talk about their problems. Intrinsic motivation, which indicates the level of self‐determinant motivation, was significantly lower in students who belonged to a sports club.

**Conclusions:**

We suggest that having parents who are medical professionals may be associated with an individual's decreased motivation when entering medical school in Japan. Though this is a novel finding, further research is needed to analyze this relationship.

## 1 INTRODUCTION

Physicians are expected to have a high level of professionalism,^1^, ^2^ which includes behaving ethically and being impartial and honest with patients. To achieve these goals, it is necessary to maintain high levels of motivation and constantly develop one's abilities. Medical school faculty members are responsible for selecting highly motivated students and training them, thereby leading to an increase in well‐trained medical professionals, and improvement of patient outcomes. According to self‐determination theory (SDT), as proposed by Ryan and Deci,^3^ medical students’ motivations for studying medicine would be based on genuine interest (intrinsic motivation) and positive valuation of the medical profession, which together are defined as autonomous motivation.^3^, ^4^, ^5^ The SDT classifies motivation into two categories: intrinsic motivation (IM) and extrinsic motivation (EM).^3^, ^6^ Autonomous motivation has previously been shown to be a preferable type of motivation that is important for student learning outcomes and positive well‐being.^4^, ^7^, ^8^, ^9^, ^10^, ^11^ The importance of maintaining high levels of motivation in medical professionals has been discussed for decades, as provider motivation has been found to affect patient outcomes, as well as their own academic performance and well‐being in medical school and their professional identity.^9^, ^12^, ^13^

Previous researches have identified various factors that impact the motivation of medical students,^7^, ^8^, ^9^, ^10^, ^11^, ^12^, ^13^, ^14^, ^15^, ^16^, ^17^ including pure interest in science, consideration of career path and income, status and prestige, family expectations, family occupation, and past medical experiences.^8^, ^15^, ^18^, ^19^ Other factors influencing motivation include receiving good grades before entering medical school,^8^, ^17^ strong recommendations from people surrounding the medical student, and admiration of familiar physician role models from life and on TV.^8^, ^9^, ^11^ However, these studies often survey participants who have already experienced numerous things as medical students or worked as doctors, and few studies have examined the correlation between motivation and background factors immediately after entering medical school, before starting medical studies.^4^, ^8^ Therefore, in this study, we identified the factors that influence motivation when entering medical school using the Academic Motivation Scale (AMS) to evaluate academic motivation, the extensively studied self‐determination theory.^20^

## 2 METHODS

### 2.1 Design

This cross‐sectional study investigated the relationship between individual backgrounds and academic motivation among medical students at their enrollment.

### 2.2 Japanese medical education

In Japan, most medical school candidates are high school students. A small number of students reenter university after having graduated from another university or after having worked for some time. In addition, Japanese medical schools have certain reserved spaces called regional quotas to secure human resources to eliminate the uneven geographical distribution of physicians. Newly graduated physicians who are a part of the regional quota are required to devote themselves to local community medicine for a set number of years.

### 2.3 Participants

University is a national university located in a relatively rural area of Japan. The average level of academic achievement of students at the time of admission at this university is similar to that of the average of all students at medical schools in Japan. One hundred and three students were freshmen in the medical school at the time immediately following admission in April 2019. The authors invited the students to complete the survey before their liberal arts class on ethics and professionalism (a class which all freshman medical students are required). The questionnaire was distributed to the class, and the students were informed that submitting the questionnaire was not mandatory and that the submission and their answers would have no effect on their grades or attendance. In addition, we explained that this was an anonymous survey and how personal information would be handled. A check box was also provided on the answer sheet of the survey to indicate nonconsent. Students were given 10 min to complete the survey. The inclusion criteria were defined as those who responded to the questionnaire and had no missing data. The exclusion criteria were defined as those who did not consent to the questionnaire, did not respond, whose response sheets could not be read by the machine, or whose responses were insufficient for analysis.

### 2.4 Sample size

The sample size (*N* = 81) was calculated by considering the following assumptions: total student number (*N* = 103), the 95% confidence level, and the margin of error (*d*  =  5%).

### 2.5 Ethical standards

We conducted this study under the condition that the information obtained would be used solely for this study, following the ICMJE requirements on privacy and informed consent from study participants and the Personal Information Protection Law in Japan. The data are completely anonymized and cannot be used to identify individuals. Therefore, after consulting with the IRB in University Hospital, we omitted approval from the Ethical board. We also explained to the participants that they were free to refuse to participate, their participation or refusal would be unknown to their instructors, and there would be no negative consequences to their standing in the program or university if they failed to participate.

### 2.6 Measures

We investigated the respondents’ backgrounds and motivations using questionnaires that were composed based on previous studies and the Japanese medical school enrollment system.^7^, ^8^, ^11^, ^15^, ^16^, ^17^, ^18^, ^19^, ^20^, ^21^, ^22^

Background factors of interest included age, gender, the experience of failing or waiting for the entrance examination, hometown, whether they gained entrance as part of a regional quota, whether they have parents who are medical professionals, scholarship and its classification on their entrance permission, extracurricular activities, smoking habits, average hours of sleep, breakfast habits, time spent working part‐time during the week, what matters in daily life, their concerns regarding campus life, and whether they have someone to talk to about their problems (Appendix S1).

We used the Academic Motivation Scale (AMS) to evaluate academic motivation, quantifying the extensively studied SDT.^14^, ^19^, ^20^ The AMS is an SDT‐based questionnaire that was developed in France to measure motivation within an academic context. It was translated into English in 1992 and is valid and appropriate for academic contexts.^20^ In the Japanese context, several papers have highlighted the high internal validity and reproducibility of the translated Japanese AMS.^22^, ^23^ Furthermore, it has been applied to international medical students not only in Europe and the United States, but also in numerous countries in the Middle East and Asia, proving its high reliability and validity.^24^, ^25^, ^26^, ^27^ The AMS classifies motivation into three categories: intrinsic motivation (IM), extrinsic motivation (EM), and amotivation. IM refers to the pleasure or satisfaction one derives from working on an object for its own sake. IM can be further divided into three categories: IM‐to‐know, IM‐to‐accomplish, and IM‐experience. IM‐to‐know refers to the pleasure one feels in learning, exploring, or trying to understand something new. IM‐to‐accomplish refers to the satisfaction one feels in trying to accomplish or create something. IM‐experience describes the state of participating in an activity to experience the stimulating sensations that result from that activity.

EM is also further divided into three categories: EM‐identified, EM‐introjected, and EM‐externally regulated. EM‐identified refers to the state in which people judge the value and importance of an object and choose it for themselves. In contrast, EM‐introjected refers to the state in which people begin to internalize the object based on past experiences, and EM‐externally regulated refers to the state in which the reason for doing something is externally controlled.

Unlike the aspects mentioned above, amotivation is a state of not finding a connection between the target and one's own behavior. The higher the scores on amotivation, the less motivation one has for the issue at hand. These motivation elements are hierarchical in SDT, with amotivation being the most non‐self‐determined state, followed by EM‐externally regulated, EM‐introjected, EM‐identified, and IM (Appendix S2).^6^ SDT states that IM and EM positively affect academic performance and well‐being, whereas high non‐self‐determined motivations, such as amotivation, have a negative impact.^12^

The AMS is divided into seven subscales (three IM, three EM, and one amotivation) as described above, comprising 28 question items. Four questions are assigned to each subscale in a random order, and each question is answered on a seven‐point scale (“1: Does not correspond at all” to “7: Corresponds exactly”). The questions in the AMS were translated into Japanese for this study. The translated Japanese questions were back‐translated using Google Translate to ensure that the content was completely understandable (March 7, 2021). A reliability test of the AMS used in this study showed that it had a Cronbach's alpha of 0.84, indicating high reliability.

### 2.7 Procedure

On May 8, 2019, the survey was administered to the medical students as described in the *Participants* section. We used a response sheet for the questionnaire and collected them after the class. The faculty of the academic affairs division used a read‐only machine to scan the response sheets. They sent the personal information in the format of a.csv file without being linked to the participants and we analyzed the data. As described above, we were allowed to omit the Ethics Committee review because only participants whose consent could be obtained were included, anonymity was ensured, there were no harmful effects anticipated, and the content was within the scope of our daily educational work to improve medical education.

### 2.8 Data analysis

Chi‐square tests and Fisher's exact tests were used to compare nominal variables. For continuous variables, *t*‐tests and Wilcoxon rank‐sum tests were used as appropriate, with Cohen's *d* being used to estimate the effect size of score differences.^28^ Regarding the selection of confounding factors for multiple logistic regression analysis, we incorporated several important factors that were likely to be significant (*p* < 0.10) on previous studies, avoiding multicollinearity: gender, age, recommended admission, receiving a scholarship, parents being medical professionals, an absence of concerns in their school life, not having someone to talk to about one's problems, and belonging to an athletic club. None of the variance inflation factor (VIF) values exceeded 10, and the mean VIF of the model was less than 1.27. All analyses were performed using the Stata statistical software, version 14.0 (Stata Corp. 2015, Stata 14 Base Reference Manual). All tests were two‐sided, with *p* < 0.05 being considered statistically significant.

## 3 RESULTS

A total of 103 medical students participated in the class (100% of the total number of students), and 96 responded (93.2% of the total number of students). Altogether, 81 questionnaires (78.6%) with consent were collected and analyzed in accordance with the exclusion criteria described previously. Table 1 shows the participant characteristics. About 40% of the students were women, and one in five were students who re‐enrolled after graduating from another school. More than 80% of the students belonged to athletic teams, and less than 40% had parents in the medical profession. More than 80% of the students had some problems with their school life; however, more than one in six students did not discuss their problems with others.

**TABLE 1:** Characteristics of participants (*N* = 81)

Characteristic	*n*	%
Gender		
Men	48	59.3
Women	33	40.7
Median age (IQR)	19 (18, 21)	
Mean age (SD)	21 (4.37)	
Experience of failing or waiting for the entrance examination		
None	32	39.5
One year	33	40.7
More than one year	13	16.1
Re‐entrance after graduating from another university	7	8.6
Re‐entrance after working	11	13.6
Duty to work at Shimane prefecture	18	22.2
Coming from Shimane prefecture	25	30.9
Received a scholarship	35	43.2
Experience with smoking	9	11.1
Belonging to an athletic club	68	84.0
Parents in the medical profession	29	35.8
Duration of sleep		
<6 h	5	6.2
6–7 h	37	45.7
7–8 h	31	38.3
>8 h	8	9.9
Breakfast habits		
≥5 days per week	63	77.8
≤4 days per week	18	22.2
Recommended admission	33	40.7
Working at a part‐time job >10 h per week	6	7.4
What is important		
Family	26	32.1
Friends	18	22.2
Grade	12	14.8
Others	25	30.9
Concerns about campus life		
Grade	29	35.8
Financial	11	13.6
Nothing	14	17.3
Other	27	33.3
A partner whom you can consult		
Friends	42	51.9
Family	19	23.5
Do not discuss with others	14	17.3
Other	6	7.4

Table 2 summarizes the results of the AMS. The mean score of amotivation was 6.84 (SD = 4.84). Among IMs, the mean score of IM‐to know was the highest. The mean score of EM‐identified, which is the most self‐identified motivation among EMs, was the highest.

**TABLE 2:** Results of the Academic Motivation Scale (*N* = 81)

	M (SD)	Median (IQR)
Amotivation	6.84 (4.85)	5 (4–8)
Total Intrinsic Motivation	55.63 (12.26)	56 (49–63)
IM‐To‐Know	22.21 (4.61)	23 (20–25)
IM‐To‐Accomplish	17.57 (5.11)	18 (14–21)
IM‐Experience	15.85 (4.77)	16 (13–19)
EM‐Identified	24.09 (4.20)	25 (22–27)
EM‐Introjected	13.67 (5.4)	14 (10–17)
EM‐Externally Regulated	19.07 (6.21)	20 (16–24)

Tables 3 and 4 show the results of the multivariate linear analysis adjusted for the total scores on amotivation and IM.

**TABLE 3:** Results of the multiple linear regression for amotivation (*N* = 81)

	*β*	SE	*t*	95% CI		*p*
Gender (Men)	0.68	1.14	0.59	−1.59	2.94	0.554
Over 24 years old	2.04	1.71	1.19	−1.37	5.46	0.236
Recommended admission	−0.79	1.22	−0.65	−3.22	1.63	0.517
Receiving a scholarship	−0.16	1.09	−0.01	−2.19	2.16	0.988
Parents in the medical profession	2.54	1.13	2.24	0.28	4.80	0.028
No concerns about campus life	0.04	1.37	0.03	−2.69	2.76	0.979
Do not discuss with others	3.28	1.40	2.35	0.49	6.07	0.022
Belonging to athletic club	3.07	1.69	1.82	−0.30	6.44	0.073

Note

Higher amotivation values indicate lower motivation.

**TABLE 4:** Results of the multiple linear regression for total intrinsic motivation (*N* = 81)

	*β*	SE	*t*	95% CI		*p*
Gender (Men)	−0.29	2.98	−0.10	−6.22	5.65	0.924
Over 24 years old	−3.28	4.49	−0.73	−12.23	5.67	0.467
Recommended admission	−1.33	3.39	−0.42	−7.69	5.02	0.677
Receiving a scholarship	2.22	2.86	0.78	−3.48	7.92	0.440
Parents in the medical profession	−4.56	2.97	−1.53	−10.48	1.37	0.129
No concerns about campus life	0.89	3.58	0.25	−6.25	8.03	0.804
Does not discuss with others	0.55	3.66	0.15	−6.76	7.85	0.882
Belonging to athletic club	−9.33	4.43	−2.11	−18.16	−0.50	0.039

Demographic factors selected for amotivation values were analyzed similarly. Only two demographic factors were significantly correlated with higher amotivation scores: having a parent in the medical profession (*β* = 2.536, 95%CI [0.277, 4.795]) and not having anyone to talk to about problems (*β* = 3.280; 95%CI [0.494, 6.067]).

Being a member of an athletic club had a significant impact on the total IM (*β* = −.329, 95%CI [−18.16, −0.497]). This result suggests that belonging to an athletic club during the first three weeks of school had a negative impact on IM. The other demographic factors were not statistically significant after adjustment.

These results suggest that belonging to an athletic club, having a parent in the medical profession, and a tendency to not talk about one's problems were correlated with the inability to maintain self‐determinant motivation. In contrast, none of the other factors, such as gender, age, retake status, hometown, or scholarship, were significantly correlated with motivation values.

### 3.1 Analysis of the factors that seem to affect AMS

Table 5 shows the comparison of AMS values for the three factors that seem to affect AMS as a result of the multivariate analysis. A comparison of the AMS scores of students who belonged to an athletic club (M = 54.22, SD = 11.78) with that of those who did not (M = 63.00, SD = 12.56) indicates a significant difference in the total value of IM (*p* = 0.041). In addition, the mean IM‐experience was significantly lower for those in the athletic group (M = 15.12, SD = 4.39) compared to those not in the nonathletic group (M = 19.69, SD = 5.02; *p* = 0.006). There were also no statistically significant differences between the other items.

**TABLE 5:** Difference in academic motivation score between students who are subject to three influential factors on academic motivation score and the other students (*N*= 81)

Parents in the medical profession	Medical Profession (*n* = 29)	Other Students (*n* = 52)	*p*‐value
	Mean (SD)	Mean (SD)	
Amotivation	8.41 (5.81)	5.96 (4.01)	0.006
Total Intrinsic Motivation	52.14 (12.87)	57.58 (11.58)	0.052
IM‐to‐know	20.83 (4.92)	22.98 (4.29)	0.017
IM‐to‐accomplish	16.59 (5.13)	18.12 (5.06)	0.127
IM‐experience	14.72 (4.43)	16.48 (4.88)	0.151
EM‐identified	20.21 (5.92)	18.44 (6.33)	0.291
EM‐introjected	14.59 (5.51)	13.15 (5.47)	0.379
EM‐externally regulated	23.34 (4.97)	24.50 (3.69)	0.352

Discussing problems with others	Not Discussed (*n* = 14)	Other Students (*n* = 67)	*p*‐value
	Mean (SD)	Mean (SD)	
Amotivation	9.50 (7.23)	6.28 (4.04)	0.039
Total Intrinsic Motivation	56.79 (12.49)	55.39 (12.29)	0.736
IM‐to‐know	22.00 (5.04)	22.25 (4.56)	0.900
IM‐to‐accomplish	18.36 (5.96)	17.40 (4.95)	0.499
IM‐experience	16.43 (4.86)	15.73 (4.78)	0.861
EM‐identified	20.64 (5.75)	18.75 (6.29)	0.263
EM‐introjected	14.29 (5.28)	13.54 (5.57)	0.822
EM‐externally regulated	22.86 (3.35)	24.34 (4.33)	0.067

Belonging to athletic club	Athletic club (*n* = 68)	Other students (*n* = 13)	*p*‐value
	Mean (SD)	Mean (SD)	
Amotivation	7.15 (5.13)	5.23 (1.77)	0.240
Total Intrinsic Motivation	54.22 (11.78)	63.00 (12.56)	0.041
IM‐To‐Know	21.84 (4.77)	24.15 (3.18)	0.201
IM‐To‐Accomplish	17.26 (5.02)	19.15 (5.46)	0.243
IM‐Experience	15.12 (4.39)	19.69 (5.02)	0.006
EM‐Identified	24.01 (4.38)	24.46 (3.23)	0.979
EM‐Introjected	13.93 (5.35)	12.31 (6.24)	0.197
EM‐Externally Regulated	19.04 (6.29)	19.23 (6.04)	0.985

Regarding the AMS for students whose parents are medical professionals, their amotivation value was significantly higher (M = 8.41, SD = 5.81) compared to the group of students whose parents are not (M = 5.96, SD = 4.01, *p* = 0.0058). IM‐to‐know scores were significantly lower in the group with parents who are medical professionals (M = 20.83, SD = 4.92) compared to those whose parents are not medical professionals (M = 22.98, SD 4.29; *p* = 0.0166). There were no significant differences between the other values.

Further, the group who did not talk about their problems with others and the group that did showed that the amotivation value was significantly higher in the group who did not discuss their problems (M = 9.50, SD = 7.23) compared to those who did not (M = 6.28, SD = 4.04; *p* = 0.039). There were no significant differences between the other values.

## 4 DISCUSSION

This study used a questionnaire survey of the background information of students and their levels of academic motivation (using the AMS) to identify and analyze factors that influence their motivation at the start of medical studies. Our primary results were threefold. First, students who belonged to an athletic club had a lower IM, especially IM‐experience, than those who did not. Second, students whose parents were medical professionals had higher amotivation and lower IM‐to‐know than students whose parents were not medical professionals. Third, students who do not discuss their problems with others had higher amotivation than students who do discuss their problems. In sum, these three factors were all associated with decreased self‐determinant motivation. In contrast to previous studies, other demographic factors, such as age and gender, had limited correlations to motivation at the time of enrollment.^13^, ^18^, ^21^

The potential reasons by which students whose parents are medical professionals have high non‐self‐determinant motivation are as follows. First, such students are expected since childhood to take over family‐owned hospitals or clinics later in life. Second, they see their parents working as medical professionals, which may lead them to experience high external motivation. Previous studies have not pointed out that low levels of motivation among medical students is linked to the fact that their parents are medical professionals. In Japan, there is a large difference between the tuition fees of public medical schools (flat payment of about 3.5 million yen for 6 years) and that of the 31 private medical schools (median: 34,096,000 yen, minimum: 19,100,000 yen, maximum: 47,365,000 yen). The parents of many students in private medical schools are doctors. Because this survey was conducted at a public university, lower non‐self‐determinant motivation may be related to the difference in parents’ financial ability; thus, a multi‐center survey is needed in the future.

Next, we examined why students who do not discuss their problems with others have high levels of non‐self‐determinant motivation. There are three possible reasons students do not tell anyone about their problems. First, they may have no one to talk to; second, they may solve their problems by themselves; and third, they cannot solve their problems, and hence, the problems accumulate. Previous studies have shown that students who lacked support from their peers tend to be depressed.^29^, ^30^ In addition, medical students with depressive tendencies have been shown to have predominantly higher amotivation, consistent with our results.^31^, ^32^ Interventions for this situation have been studied, and we hope that early detection and intervention can improve medical students’ motivation.^33^

Furthermore, we discuss the possible cause of the low self‐determinant motivation of students belonging to an athletic club. It has been shown that exercise can improve academic performance and mental health.^34^, ^35^, ^36^, ^37^, ^38^ However, few previous studies have described the relationship between belonging to an athletic club and motivation at the time of entrance. In addition, when the students have joined an athletic club just after entering school, the effects of exercise, as shown in previous studies, may not be obtained. The “act” of belonging to an athletic club may be correlated with motivation. To the best of our knowledge, no previous studies have shown a correlation between belonging to an athletic club at the time of admission and motivation or academic performance of medical school students in Japan. Thus, we believe this to be a new finding regarding Japanese medical students immediately after admission.

### 4.1 Limitations

There are several limitations to this study that should be considered. First, as a single‐center study in Japan, the current study cannot be generalized to medical students in all countries. In a systematic review on motivation of medical students reported by Goel et al., motivation was divided into the scientific domain (interest in medicine, intellectual curiosity, willingness to do research, etc.), societal domain (job stability, high income, status, and suggestions from parents), and humanitarian domain (wanting to contribute to others or the community and country, wanting to help the others).^8^ According to this review, studies of motivation among medical students in high‐income countries such as Japan and the United States tended to show a high level of factors referring to the scientific domain and a low level of societal and humanitarian factors. On the other hand, motivations within the scientific domain were the least common in middle‐income and lower‐middle‐income countries. Thus, the economic stability of the country where the medical student is studying, and their social and cultural backgrounds (e.g., religion) likely influence their motivations.

Another limitation to the current study is its limited sample size. Although there was a high response rate for the survey at the single site, we ideally should have collected the data for multiple school years in order to collect more responses. Also, for a variety of unavoidable reasons, we were not able to collect data in person for this pilot study; however, we plan to conduct similar evaluations at multiple sites and in various schools in the future.

A third limitation is that we could not specify the participants parents’ occupations, and instead could only gather whether their occupation was in the medical field; thus, results may differ depending on the type of medical role the parent occupies. For example, in Japan, the influence of physician–parents may affect other factors, such as the student's financial status and if/how the parents encourage their child to become a doctor.^4^

Finally, the AMS using in this study was a Japanese translation of the English questionnaire, and limited scientific evidence exists to indicate whether the AMS is valid when used in the Japanese education system.^22^, ^23^ One previous paper did conclude that the Japanese version was internally valid and reproducible^22^; however, to the best of our knowledge, this is the first study conducted in Japan using the AMS to assess medical students’ motivation at the time of admission, and we believe the current study is fundamental as a pilot study in Japan.

## 5 CONCLUSIONS

Our study showed that students whose parents are medical professionals and those who do not discuss their problems with others typically have lower motivation when admitted to medical school. Further research is needed to determine whether these results apply to other universities and countries, and to clarify factors that influence motivation at the time of medical school enrollment.

## CONFLICT OF INTEREST

The authors declare that they have no competing interests.

## AUTHOR CONTRIBUTIONS

NN and TW designed the study, the main conceptual ideas, and the proof outline. NN analyzed all the data. KK supported the writing of the manuscript, and KO supported data collection. TW supervised all the processes. All authors discussed the results and commented on the manuscript. All authors read and approved the final manuscript.

## CONSENT TO PARTICIPATE

We obtained the informed consent from the participants under the condition that the information obtained would be used solely for this study, in accordance with the ICMJE requirements on privacy and informed consent from study participants and the Personal Information Protection Law in Japan, and with the Helsinki Declaration of 1965, as revised in 2013 in Brazil.

## Supporting information

Appendix S1

Appendix S2
