# Supplementary material for: Background factors associated with academic motivation for attending medical school immediately after admission in Japan: A single‐center study
Source: J Gen Fam Med. 2022 Feb 16;23(3):164–71. doi: 10.1002/jgf2.528 (PMC9062539; doi:10.1002/jgf2.528)
Supplement: Supplementary file 1 — Appendix S1 [file JGF2-23-164-s002.docx]

# Appendix S1: Questionnaire

Please complete Sheet 1 below.

The results of this questionnaire will in no way be detrimental to your academic performance or promotion. Please mark only one answer for each mark column as you see fit.

Mark Column 1. Please mark the appropriate department.

① Medical Science ② Nursing

Mark Column 2, 3. Please mark your age.

Mark the tens digit on 2 and the ones digit on 3.

Mark Column 4. Please mark your gender.

① Male ② Female

Mark Column 5. Please choose the appropriate category about your experience of failing or waiting for the entrance examination or re-entrance.

① None ② One year ③ Two years ④ More than two years

⑤ Re-entrance (after dropping out of other universities) ⑥ Re-entrance (after graduating from other universities) ⑦ Re-entrance (after working)

Mark Column 6. Please choose your hometown.

① Shimane Prefecture ② Other

Mark Column 7. Please choose the appropriate box for whether or not you are enrolled in the regional quota system.

① Enrolled in the regional quota system ② Not enrolled

Mark Column 8. About your parents’ occupation:

① Both parents or one parent is a medical professional. ② Both parents are non-medical professionals.

Mark Column 9. Please mark the appropriate box for the existence and type of scholarship.

① Scholarships from the Japan Student Services Organization (no interest)

② Scholarships from the Japan Student Services Organization (with interest) ③ Scholarships from the local government ④ Scholarships from specific hospitals ⑤ Use of other scholarships ⑥ Not receiving any scholarships

Mark Column 10. Please mark one that applies regarding club activities.

① Athletic club ② Cultural club ③ Athletic circle ④ Cultural circle ⑤ Other ⑥ Have not decided yet ⑦ No affiliation

Mark Column 11. Please mark the appropriate category about smoking experience.

① Never smoked before ② Currently smoking ③ Have smoked in the past

Mark Column 12. Mark the average number of hours of sleep per day (if more than 10 hours, mark 0).

Mark Column 13. Please mark the appropriate category about your breakfast habits.

① Eat every day ② 5–6 times a week ③ 3–4 times a week ④ 1–2 times a week ⑤ Do not eat

Mark Column 14. Please mark your admission category.

① Recommended Admissions ② Not recommended Admissions

Mark Columns 15, 16. Please mark the amount of time you spend working part-time during the week.

Mark the tens digit on 15 (if you have one digit or zero hours, mark 0) and the ones digit on 16.

Mark Column 17. Please mark the most important things in your life.

① Family ② Friends ③ Lover ④ Club activities ⑤ Part-time job ⑥ Academic performance ⑦ Hobbies ⑧ Others ⑨ Nothing in particular is important

Mark Column 18. Please mark the choice for your greatest concern.

① Friendships ② Relationships with seniors and juniors ③ Family relationships ④ Romantic relationships ⑤ Academic performance ⑥ Future career path ⑦ Financial situation ⑧ Others ⑨ No particular concerns

Mark Column 19. Please mark the category of whether or not you have someone to talk to if you have problems.

① Friends ② Seniors and juniors ③ Family ④ Teachers ⑤ Counselors ⑥ No one to talk to ⑦ Do not discuss with others ⑧ I do not have any problems to begin with

Please complete Mark Sheet 2 below.

The results of this questionnaire will in no way be detrimental to your academic performance or promotion.

Please complete as you see fit.

The following are answers to the question, ‘Why did you go to college?’

Please mark ‘1: Does not correspond at all’ to ‘7: Corresponds exactly’.

1. Because with only a high-school degree I would not find a high-paying job later on.

2. Because I experience pleasure and satisfaction while learning new things.

3. Because I think that a college education will help me better prepare for the career I have chosen.

4. For the intense feelings I experience when I am communicating my own ideas to others.

5. Honestly, I do not know; I really feel that I am wasting my time in school.

6. For the pleasure I experience while surpassing myself in my studies.

7. To prove to myself that I am capable of completing my college degree.

8. To obtain a more prestigious job later on.

9. For the pleasure I experience when I discover new things never seen before.

10. Because eventually it will enable me to enter the job market in a field that I like.

11. For the pleasure that I experience when I read interesting authors.

12. I once had good reasons for going to college; however, now I wonder whether I should continue.

13. For the pleasure that I experience while I am surpassing myself in one of my personal accomplishments.

14. Because when I succeed in college, I feel important.

15. Because I want to have ‘the good life’ later on.

16. For the pleasure that I experience in broadening my knowledge about subjects that appeal to me.

17. Because this will help me make a better choice regarding my career orientation.

18. For the pleasure that I experience when I feel completely absorbed by what certain authors have written.

19. I cannot see why I go to college and frankly, I could not care less.

20. For the satisfaction I feel when I am in the process of accomplishing difficult academic activities.

21. To show myself that I am an intelligent person.

22. To have a better salary later on.

23. Because my studies allow me to continue to learn about many things that interest me.

24. Because I believe that a few additional years of education will improve my competence as a worker.

25. For the ‘high’ feeling that I experience while reading about various interesting subjects.

26. I do not know; I cannot understand what I am doing in school.

27. Because college allows me to experience a personal satisfaction in my quest for excellence in my studies.

28. Because I want to show myself that I can succeed in my studies.

**Appendix S2** Classification of motivation in the Self-Determination Theory
