# Supplementary figures and images for: Background factors associated with academic motivation for attending medical school immediately after admission in Japan: A single‐center study
Source: J Gen Fam Med. 2022 Feb 16;23(3):164–71. doi: 10.1002/jgf2.528 (PMC9062539; doi:10.1002/jgf2.528)

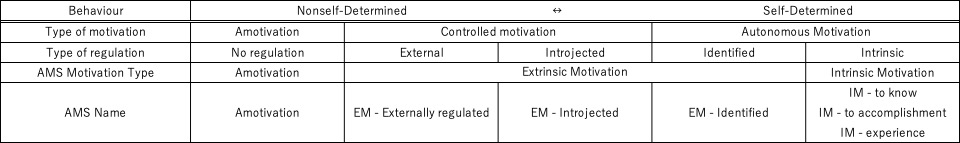

Supplement: Supplementary file 2 — Appendix S2 [file JGF2-23-164-s001.jpeg]
